# Some Misdirected Letters

**Published:** 1894-05

**Authors:** 


### SOME MISDIRECTED LETTERS.

The following form of letter has been distributed, we know not how widely, in Georgia, Alabama, and perhaps other Southern States:

RICHMOND, VA., April 18th, 1894.

DEAR DR. ———: Please spare a moment to enclosed circular. You see this is the representative Southern medical journal, adopted even by the profession of D. C., E. Tenn., etc. Your State has no journal, although this journal has many subscribers there. Won't you help to further develop it? If you promptly remit a dollar and a half, or if you subscribe now to pay two dollars by next fall, I will send journal one year, beginning this month. Isn't that liberal enough?

Yours very truly, ——— ———.

The first copy which we saw was sent us by one of our subscribers in Southern Georgia, along with a very emphatic protest against the statement that Georgia has no medical journal. The ethics of medical journalism were naturally somewhat strained by such enterprising correspondence, so we forwarded the letter to our amiable Virginia contemporary, along with appropriate comments of our own. We were pleased to learn from the editor's reply that it was by mistake of his typewriter that such letters were sent into Georgia and other Southern States having medical journals.

With this explanation we hesitated to speak of the matter at all, but justice to ourselves and fairness to our contemporary seem to require this much.

---

### MODERN HOMEOPATHY.

A late issue of the *North American Journal of Homeopathy*, a representative journal of its class, contains a tearful lament over the present status of homeopathic medicine. Our brethren of the infinitesimals will find very little comfort in such a confession of the truth. We quote:

The homeopathic profession has some lessons to learn, and it needs to learn them quickly. In these latter days of grace the public is noting that in public affairs the homeopathic school is invisible or nearly so. It expresses no opinions on sanitary matters, it solves no hygienic problems, it gives no public instruction, it is interested in few public institutions, in short, it has little as a school to do with public medicine. Thirty years ago there was public spirit enough manifested; there were giants in those days. But now there is stagnation and there is scarcely a ripple to disturb the placid surface of affairs.

---

THE AMERICAN MEDICAL ASSOCIATION will meet in San Francisco, June 5th. The Association will assemble in Odd Fellows Hall, corner of Market and Seventh streets. The headquarters of the Association will be at the Palace hotel, corner of Market and Montgomery streets. The round trip rates will be: $65.50 from Missouri river points, Sioux City, Omaha, Leavenworth, Kansas City, etc., and $77.50 from St. Louis, Memphis, New Orleans, etc. All tickets sold at these points carry five coupons of admittance to the Midwinter Fair which will be continued until June 30th.

---

WE have received the last volume (Vol. VI.) of the Transactions of the Southern Surgical and Gynecological Association, for the meeting held in New Orleans, November, 1893. The volume is somewhat larger than its predecessors and contains many excellent papers. To the zeal of the Secretary, Dr. Davis, of Birmingham, is due much of the success of the Association, as well as the attractiveness of the published transactions.

